# Instruments for assessing insight in psychosis: A systematic review of psychometric properties

**DOI:** 10.1017/S0033291725101918

**Published:** 2025-11-26

**Authors:** Hadar Hazan, Sümeyra N. Tayfur, Sneha Karmani, Tony Gibbs-Dean, Catalina Mourgues, Vinod Srihari

**Affiliations:** 1 Community & Behavioral Health Program, SUNY Polytechnic Institute, Utica, NY, USA; 2 Department of Psychiatry, Yale University School of Medicine, New Haven, CT, USA

**Keywords:** COSMIN, insight, measurement, psychometrics, psychosis, systematic review

## Abstract

**Background:**

Insight into psychosis is a multidimensional construct involving awareness of illness, attribution of symptoms, and perceived need for treatment. Despite extensive research, substantial variability in how insight is conceptualized and measured continues to hinder clinical assessment and cross-study comparisons.

**Methods:**

Following Preferred Reporting Items for Systematic Review and Meta-Analysis Protocols guidelines and a registered International Prospective Register of Systematic Reviews protocol (CRD42024558386), we conducted a systematic search across five databases (*n* = 2,184). Twenty-nine studies met the inclusion criteria, comprising 15 primary scale development papers and 10 independent validation studies. We included instruments explicitly designed to assess insight in schizophrenia-spectrum, and evaluated them using the COnsensus-based Standards for the selection of health Measurement INstruments Risk of Bias checklist. Psychometric domains assessed included content validity, structural validity, construct validity, criterion validity, internal consistency, reliability, responsiveness, and interpretability.

**Results:**

Fifteen distinct insight scales were identified, comprising nine clinician-rated instruments, five self-report tools, and one hybrid format. Most demonstrated adequate content and structural validity, with 11 achieving ‘very good’ reliability ratings. Four scales showed the strongest overall psychometric support. However, responsiveness to clinical change was rarely tested, and cross-cultural validation remained limited. Earlier instruments primarily emphasized clinician-rated illness awareness, whereas more recent tools incorporated cognitive, neurocognitive, and subjective dimensions. Discrepancies between self-report and clinician ratings were common and often clinically meaningful. These findings underscore the need for multidimensional, psychometrically robust, and context-sensitive tools to advance both clinical assessment and research on insight in psychotic disorders.

## Introduction

Insight into psychosis is a complex and multidimensional concept, encompassing a patient’s awareness of their mental illness and its implications (Chakraborty & Basu, 2010). This awareness, or lack thereof, is closely tied to critical outcomes, including treatment adherence, prognosis, and quality of life (Goldberg, Green-Paden, Lehman, & Gold, 2001; Lincoln, Lüllmann, & Rief, 2007). Historically, a lack of insight was considered a hallmark of psychosis, as early definitions of insanity centered on delusional cognition (Lewis, 1934). However, this view began to change with the mid-nineteenth century’s intellectual shift toward self-awareness and introspection (Berrios & Marková, 2004). Concepts such as consciousness and subjective experience allowed for a broader understanding, paving the way for recognizing the possibility of individuals gaining insight into their mental illness (Berrios, 1994).

The nature of insight has challenged scholars aiming to define and conceptualize it in psychosis (Kamens et al., 2025). Aubrey Lewis’s (1934) essay, *The Psychopathology of Insight*, marked a key moment in this endeavor, describing insight as the ‘correct attitude to a morbid change in oneself’ (p. 332). Lewis acknowledged the complexity of the term, noting the need for further exploration; however, systematic investigations into insight remained scarce for much of the twentieth century. Early approaches relied heavily on anecdotal and impressionistic methods (Appelbaum, Mirkin, & Bateman, 1981; Eskey, 1958; Soskis & Bowers, 1969; Van Putten, Crumpton, & Yale, 1976; Whitman & Duffey, 1963), which lacked the standardization required for clinical application (see Supplementary Appendix 1 for a review of these methods). This gap underscored the pressing need for structured and reliable assessment tools.

The introduction of structured insight scales in the late 1980s marked a turning point in the field (Davidhizar, 1987; McEvoy, Appelbaum, Apperson, Geller, & Freter, 1989). These early tools primarily focused on clinical insight, typically defined as awareness of having a mental illness and the need for treatment. They employed diverse methodologies, ranging from binary formats (e.g. yes/no; Marková & Berrios, 1992) to spectrum-based ratings (e.g. 0–3; Birchwood et al., 1994), and included both self-report (Birchwood et al., 1994) and clinician-rated assessments (Amador et al., 1993a). Conceptualizations of insight in these scales often spanned dimensions such as illness recognition (McEvoy, Appelbaum, et al., 1989), treatment adherence (McEvoy, Appelbaum, et al., 1989), symptom relabeling (Amador & Strauss, 1993b), and awareness of social, personal, and internal changes (Marková et al., 2003; Marková & Berrios, 1992). More recent instruments have expanded the scope of insight assessment to include cognitive and neurocognitive domains, as exemplified by the Beck Cognitive Insight Scale (BCIS; Beck, Baruch, Balter, Steer, & Warman, 2004) and the Measure of Insight into Cognition (MIC) (Saperstein, Thysen, & Medalia, 2012). These measures reflect a broader and more nuanced understanding of self-awareness in psychosis. Importantly, cognitive insight, as conceptualized by Beck, is distinct from traditional clinical insight: it reflects a metacognitive style characterized by self-reflectiveness and openness to corrective feedback, and it can be applied to both clinical and nonclinical populations to assess cognitive style rather than illness awareness (Donohoe et al., 2009). Although cognitive insight is sometimes situated within the broader framework of metacognition, the two are conceptually distinct: metacognition refers to higher-order processes of reflecting on mental states in general, whereas cognitive insight specifically captures the evaluation of one’s own potentially distorted beliefs.

Several valuable studies have advanced the understanding of insight in psychosis through longitudinal and intervention designs (e.g. David, Bedford, Wiffen, & Gilleen, 2012; Rathod, Kingdon, Smith, & Turkington, 2005; Wiffen, Raballo, & David, 2010), culturally diverse cohorts (Saravanan et al., 2010), neurocognitive and neuroanatomical correlates (Morgan et al., 2010), comparative evaluation of multiple insight measures (Sanz, Constable, Lopez-Ibor, & Kemp, 1998), and examination of awareness domains across self- and clinician-administered formats (Gilleen, Greenwood, & David, 2011). These works provide important contributions to the field, including evidence on responsiveness to treatment, cultural variability, and conceptual distinctions within the construct of insight. However, their primary focus is not on the systematic evaluation of the psychometric properties of insight scales themselves, which remains the central aim of the present review.

This variability in operational definitions, as well as the methodological issues (Goldberg et al., 2001) related to reliability, validity, and psychometric robustness (Ghaemi & Pope, 1994; Amador & David, 2004; Mintz, Dobson, & Romney, 2003), continue to complicate standardization and limit the generalizability of findings. Additionally, challenges like small and heterogeneous samples further complicate the interpretation of research findings. This article addresses these challenges by systematically reviewing existing structured insight scales, focusing on their design, purpose, and psychometric properties using the COSMIN (COnsensus-based Standards for the selection of health Measurement INstruments) framework (Mokkink et al., 2010). Our aim is to provide a comprehensive overview of existing insight scales used in psychotic disorders; highlight their similarities, differences, psychometric properties, strengths, and limitations; and inform researchers and clinicians who must select the most appropriate instrument for their specific purposes.

## Methods

### Literature search

We conducted this systematic review following the Preferred Reporting Items for Systematic Review and Meta-Analysis Protocols (PRISMA-P) guidelines (Moher et al., 2015). A protocol for this review was registered in the International Prospective Register of Systematic Reviews (PROSPERO) (registration no: CRD42024558386) and published elsewhere (Hazan, Funaro, & Srihari, 2025). Our search strategy included five electronic databases: MEDLINE, APA PsycInfo, Embase, Web of Science Core Collection, and Health and Psychosocial Instruments Database (HaPI). We used various related terms such as ‘insight’, ‘awareness’, ‘illness awareness’, ‘illness attitudes’, and ‘illness beliefs’. These were combined with terms pertaining to diagnoses or disorders, including ‘Schizophrenia Spectrum and Other Psychotic Disorders’, ‘psychosis’, ‘psychoses’, ‘psychotic’, and ‘schizo’. Additionally, we included terms related to insight assessments, including ‘assessment’, ‘checklist’, ‘interview psychological’, ‘instrument’, ‘inventory’, ‘measure’, ‘questionnaire’, ‘scale’, ‘score’, ‘survey’, ‘test’, ‘tests’, and ‘tool’, ‘surveys and questionnaires’ (see Supplementary Appendix 2 for a sample search strategy).

### Inclusion and exclusion criteria

To be included in this review, studies must meet the inclusion criteria, defined according to the COSMIN guidelines:

*Construct of interest*

- Inclusion: Studies evaluating instruments designed to measure the construct of insight in psychosis. This includes instruments assessing awareness of illness, provided the concept is directly linked to insight within the context of psychosis.

*Population of interest*

- Inclusion: Studies involving individuals diagnosed with schizophrenia spectrum disorders, including schizophrenia, schizoaffective disorder, or other psychosis-related diagnoses.
- Exclusion: Studies focusing on populations without a diagnosis of schizophrenia spectrum disorder or related mental health conditions.

*Type of measurement instrument*

- Inclusion: Studies investigating measurement instruments such as self-report tools, observer ratings, behavioral assessments, multidimensional scales, or semi-structured interviews that measure insight.
- Exclusion: Studies evaluating instruments not related to insight measurement or those focused exclusively on non-measurement-based interventions.

*Measurement properties*

**Inclusion:** Studies were required to explicitly aim to evaluate at least one psychometric property of an insight measure. Eligible properties included:

- Reliability (*internal consistency, test–retest reliability, inter-rater reliability*),
- Validity (*content, structural, construct, or criterion validity*), and
- *Responsiveness, interpretability, or clinical utility* (evidence of sensitivity to change or feasibility in applied settings).

**Inclusion criteria**: Studies were included if they reported at least one of the following psychometric properties:

- Reliability: Including internal consistency, test–retest reliability, or inter-rater reliability.
- Validity: Including
  - Content validity (relevance and comprehensiveness of items),
  - Structural validity (dimensional structure evaluated through factor analysis),
  - Construct validity (evidence based on hypothesis testing, such as correlations with related measures or known-groups comparisons), and
  - Criterion validity (comparison with an external gold standard, when available).
- Responsiveness, interpretability, or clinical utility: Including evidence that the instrument detects meaningful change over time or is feasible for use in applied settings.

**Exclusion criteria**: Studies that lack psychometric data or do not report on any of the specified measurement properties.

*Eligible study types*

- Inclusion:
  - Primary studies published in peer-reviewed journals.
  - Studies explicitly aiming to evaluate the use of an existing measurement instrument or develop a new one in alignment with COSMIN guidelines.
  - Full-text articles published in English.

### Literature selection

**Figure 1.**
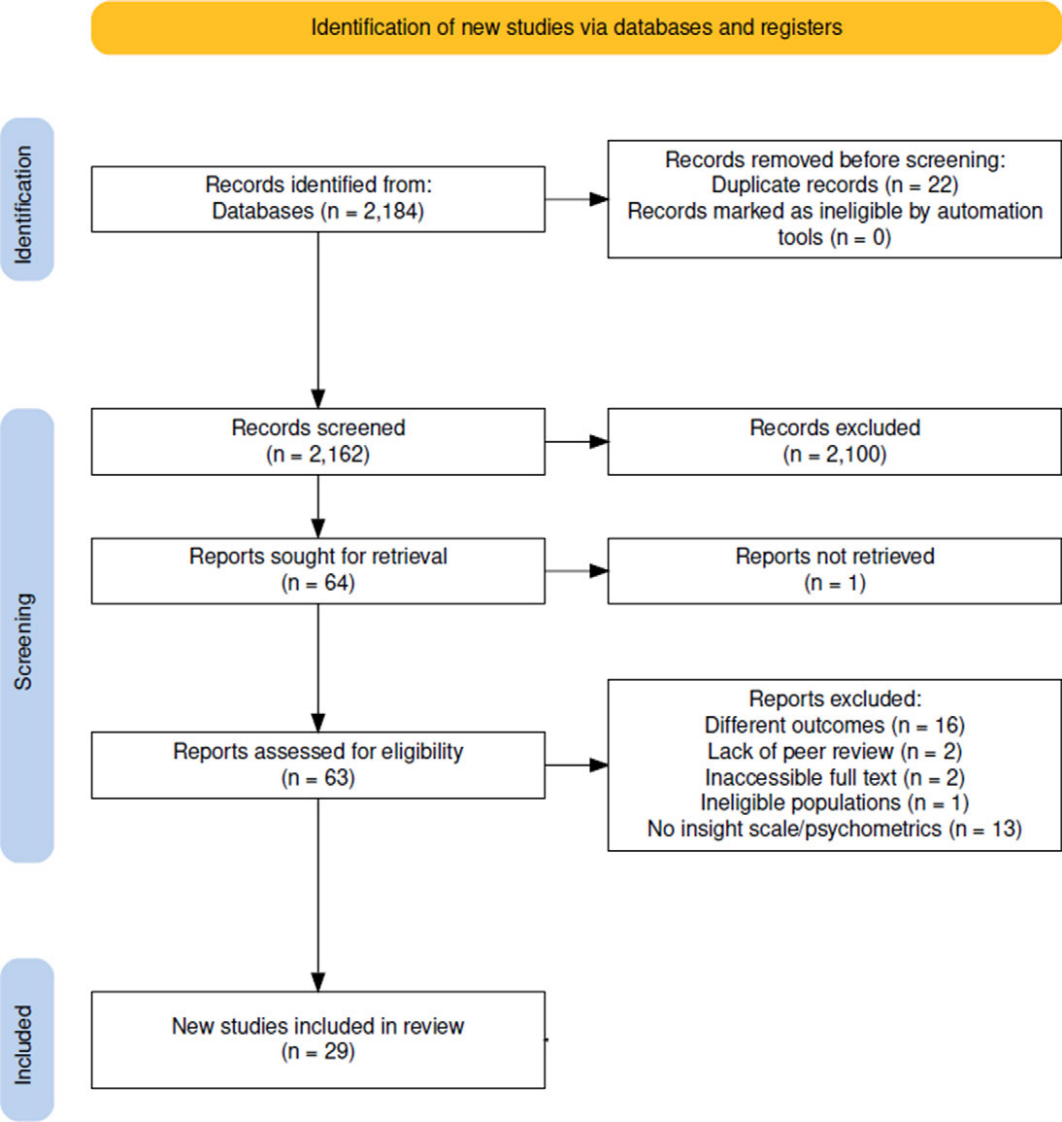
PRISMA flowchart detailing the study selection process.

A comprehensive search was conducted in five electronic databases**:** Ovid MEDLINE ALL, APA PsycINFO (Ovid), Embase (Ovid), Web of Science Core Collection (Clarivate), and HaPI (Ovid). The search results were imported into *Covidence* (Covidence systematic review software, 2024), a systematic review management tool, where duplicates were automatically removed. Title and abstract screening were carried out independently by two reviewers (HH and SK), adhering to the inclusion and exclusion criteria specified in the study protocol. Full-text screening was conducted by two reviewers (HH and ST) to evaluate all potentially eligible articles, with pilot screening conducted beforehand to standardize the criteria application. Reference lists of included articles were manually reviewed to identify any additional eligible studies. Discrepancies were resolved through consensus or, when necessary, consultation with a third reviewer (VHS). Once consensus was reached on the eligible studies, the first author and a second reviewer collaboratively extracted data from each included study and assessed its psychometric properties using the COSMIN framework. [A detailed PRISMA flowchart (Figure 1) outlines the identification, screening, and inclusion process, and the corresponding PRISMA checklist is provided in Supplementary Appendix 3]. To supplement COSMIN-based assessments, we incorporated findings from key empirical studies that evaluated psychometric characteristics of insight scales, particularly where primary scale development studies were unavailable or incomplete. Supporting studies are summarized in Table 2.

## Results

### Identification and selection of insight scales

The initial database search yielded 2,184 records. Following the removal of 22 duplicate entries, 2,162 titles and abstracts were screened. Of these, 2,100 records were excluded for not meeting the inclusion criteria. Sixty-four full-text articles were sought for retrieval, of which one could not be obtained. A total of 63 articles were assessed for eligibility, resulting in the exclusion of 34 articles due to different outcomes (*n* = 16), lack of peer review (*n* = 2), inaccessible full text (*n* = 2), ineligible populations (*n* = 1), and absence of insight scale development or psychometric evaluation (*n* = 13). Twenty-nine studies met the inclusion criteria and were included in the review (see Figure 1).

Fifteen *distinct* insight scales were retained from 28 studies, including 15 primary scale development papers and 10 independent psychometric evaluations. Three instruments with two published versions – the Schedule for the Assessment of Insight (SAI) and its expanded version (SAI-E), the Marková Insight Scale (IS) and later revised (IS-R), and the MIC clinician-rated (MIC-CR) and MIC self-report (MIC-SR) – were counted as single scales, despite being reported in separate publications, to reflect their conceptual continuity and shared measurement framework. A complete list of the 28 papers is provided in Supplementary Appendix 4.

Most instruments originated in the United States and the United Kingdom, with additional contributions from Austria, Canada, Poland, and Taiwan. Sample sizes ranged from 17 to 425. Studies were conducted in a range of settings, including inpatient psychiatric units, outpatient clinics, and community mental health services, offering diverse clinical contexts for insight scale validation. Table 1 summarizes key characteristics of the 15 included insight scales, including authorship, country of origin, assessment type (clinician-rated or self-report), primary aim, sample size, number of items, assessed insight domains, and scoring methods.

**Table 1.** Descriptive characteristics of insight scales

Scale	Author(s)	Year	Country	Type	Aim	*N*	Items	Insight domains	Scoring description
Davidhizar	Davidhizar et al.	1986	USA	Self-report	Relationship between insight and treatment	50	10	Beliefs, illness awareness, treatment attitudes	5-point Likert (1–5)
ITAQ	McEvoy et al.	1989	USA	Clinician-rated	Illness recognition and need for care	52	11	Illness recognition, care necessity	3-point scale (0–2)
SAI/SAI-E	David et al.	1990/1992	UK	Clinician-rated	Multidimensional insight structure	91	7	Awareness, relabeling, compliance	Domain scores (0–4, 0–6); total: 14–18
MIS/MIS-R	Marková et al.	1992/2003	UK	Clinician/self	Subjective insight and change over time	43/64	32/30	Awareness, internal change, attributions	Dichotomous (0/1); total score varies
SUMD	Amador et al.	1993a	USA	Clinician-rated	Awareness and attribution, current/past symptoms	43	6+	Illness, symptoms, consequences	5-point ordinal, hierarchical
BIS	Birchwood et al.	1994	UK	Self-report	Brief self-rated multidimensional insight	133	8	Awareness, relabeling, treatment need	Subscale + total score (0–12)
AII	Cuffel et al.	1996	USA	Clinician-rated	Awareness and outpatient adherence prediction	89	7	Illness recognition, treatment need	5-point ordinal
SALI	Dębowska et al.	1998	Poland	Clinician-rated	Insight into treatment and recurrence	61	≥6	Treatment acceptance, efficacy, recurrence	3-point ordinal (1 = good, 3 = poor)
SAIQ	Marks et al.	2000	USA	Self-report	Cognitive-affective illness beliefs	59	17	Beliefs, treatment need, outcome worry	4-point Likert
SIP	Yen et al.	2001	Taiwan	Clinician-rated	Multidimensional insight assessment	100	9	Symptoms, relapse, etiology, treatment, impact	4-point ordinal (1–4)
Insight Index	Lang et al.	2003	Austria	Mixed (3 self-rated items, 1 clinician-rated item)	A brief and practical index of insight and its demographic and clinical predictors	425	4	Awareness of illness, knowledge of diagnosis/symptoms, treatment expectations, and interviewer-rated global insight	1 point for each domain (total score: 0–4). Insight levels: 0–1 = very poor, 2 = poor, 3 = moderate, 4 = good
BCIS^a^	Beck et al.	2004	USA	Self-report	Metacognitive insight (cognitive style)	150	15	Self-reflectiveness, certainty	Composite index: SR – SC
MIC^a^	Medalia & Thysen	2008	USA	Clinician-rated	Awareness of cognitive deficits	75	12	Attention, memory, executive function	5-point ordinal, with attribution
	Medalia et al.			Self-report	Self-perceived cognitive problems	215	12	Same as MIC-CR	0–3 scale (frequency); total 0–36
EIS	Tranulis et al.	2008a	Canada	Clinician-rated	Narrative insight across perspectives	54	9	Awareness, attribution, social impact	Coded dichotomously
VAGUS	Gerretsen et al.	2014	Canada	Both formats	Clinician and self-report insight measure	215/140	10/5	Illness awareness, attribution, treatment need	10-point Likert per item

a Cognitive insight.

### Description of insight scales

#### Early measures (1980s–1990s)

Initial efforts to operationalize the concept of insight as a multidimensional construct began with Davidhizar, Austin, and McBride (1986), who developed a 10-item self-report scale capturing *beliefs about symptoms*, *awareness of illness*, *perceived vulnerability to relapse*, and *attitudes toward treatment*, with strong internal consistency (*α* = 0.90).

McEvoy, Appelbaum, et al. (1989) introduced the Insight and Treatment Attitudes Questionnaire (ITAQ), a clinician-rated instrument focusing on *illness recognition* and the *perceived need for psychiatric care*, notable for its predictive value regarding treatment adherence but limited responsiveness to symptom change.

David (1990) and David, Buchanan, Reed, and Almeida (1992) formalized a three-component model – *illness awareness*, *symptom relabeling*, and *treatment compliance* – through the development of the SAI and SAI-E, establishing early structural validity and known-groups discriminant validity.

Marková and Berrios (1992) advanced the field by emphasizing the subjective experience of insight in the Preliminary Insight Scale (IS) and IS-R to refine its measurement of *awareness of various changes* happening within an individual and their *perception of the environment.* Building on the notion of insight as multidimensional, Amador et al. (1993a) introduced the Scale to Assess Unawareness of Mental Disorder (SUMD), a comprehensive clinician-rated tool assessing awareness and attribution across multiple symptom domains over time, with high interrater reliability.

Birchwood et al. (1994) developed the Birchwood Insight Scale (BIS), a brief self-report measure targeting *illness awareness*, *treatment need*, and *symptom relabeling*, and demonstrated its sensitivity to clinical recovery. Subsequently, the Awareness of Illness Interview (AII; Cuffel, Alford, Fischer, & Owen, 1996) and the Scale to Assess Lack of Insight (SALI; Dębowska, Grzywa, & Kucharska-Pietura, 1998) provided additional brief, clinician-rated formats focused primarily on treatment-related insight, with the AII emphasizing outpatient adherence prediction and the SALI correlating insight deficits with symptom severity.

#### Contemporary measures (2000s–2010**s)**

Newer scales broadened the conceptualization of insight to encompass affective, cognitive, and functional domains. The Self-Appraisal of Illness Questionnaire (SAIQ; Marks, Fastenau, Lysaker, & Bond, 2000) extended measurement beyond illness acknowledgment to include *beliefs about outcomes* and *worry related to the illness.* The BCIS (Beck et al., 2004) redefined insight through a metacognitive lens, emphasizing self-reflectiveness and overconfidence in judgments, and demonstrated distinctiveness from clinical insight.

The MIC – both MIC-CR (Medalia & Thysen, 2008) and MIC-SR formats (Medalia, Saperstein, & Revheim, 2008) – introduced the assessment of neurocognitive insight, evaluating awareness of deficits in attention, memory, and executive functioning. The Extracted Insight Scale (EIS; Tranulis, Corin, & Kirmayer, 2008a) uniquely captured narrative constructions of insight by integrating perspectives from patients, family members, and clinicians. Finally, the VAGUS Insight into Psychosis Scale (Gerretsen, Chakravarty, Mamo, et al., 2014) provided parallel clinician- and self-report versions, with strong reliability, validity, and sensitivity across core insight dimensions, enhancing its clinical and research utility.

#### Psychometric characteristics of insight scales

**Figure 2.**
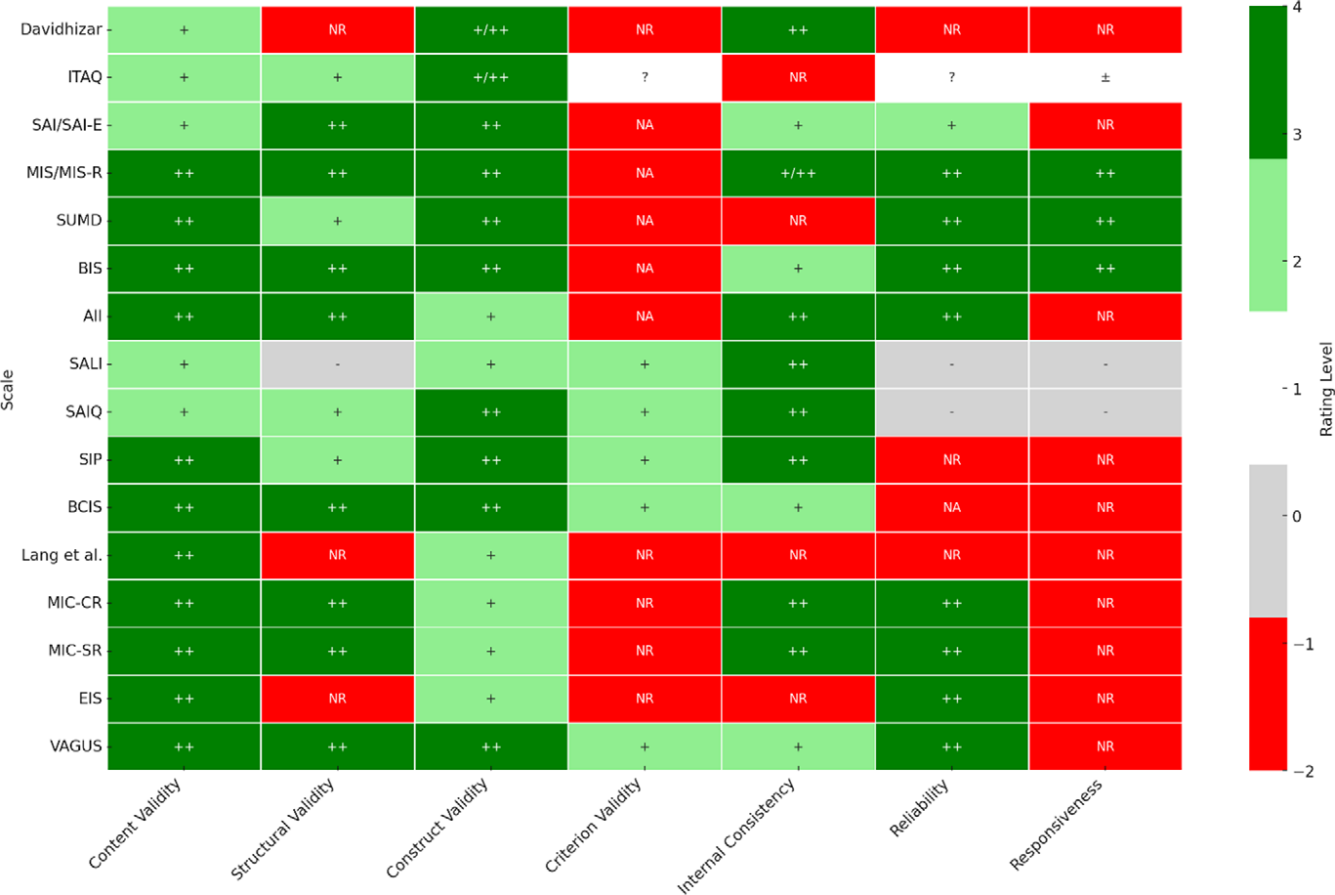
Psychometric properties of insight scales (COSMIN ratings). *Note:* +, ‘adequate’; ++, ‘very good’; ±, ‘doubtful’; −, ‘inadequate’; ?, ‘indeterminate’; NA, ‘not applicable’; NR, ‘not reported’.

Psychometric properties were evaluated according to COSMIN guidelines across seven domains. *Content validity* assessed whether each scale adequately represented key insight constructs based on theory, expert review, or patient feedback. *Structural validity* refers to the underlying internal structure of scales, which is evaluated through factor analysis or related methods. *Construct validity* examined associations between scale scores and related clinical or functional measures. *Criterion validity* assessed agreement with external standards or previously validated instruments. *Internal consistency* measures the correlation between items within a scale, typically through Cronbach’s alpha. *Reliability* refers to the consistency of scales tested by test–retest stability or inter-rater agreement. *Responsiveness* evaluated an instrument’s sensitivity to clinical or symptom-related change over time. Figure 2 summarizes the COSMIN ratings (see Supplementary Appendix 5 for detailed psychometric properties of scales).

#### Content validity

Most scales were grounded in theory and clinical experience. Strong content development processes were reported for the SUMD, Marková Insight Scale (MIS)/Marková Insight Scale–Revised (MIS-R) (MIS/MIS-R), AII, MIC, and VAGUS, which were based on multidimensional insight models or pilot testing. Other instruments, such as the Davidhizar scale, SALI, and SAIQ, lacked patient involvement or qualitative development work.

#### Structural validity

Structural validity was established for many scales through exploratory or principal component analysis. The MIS/MIS-R demonstrated a multidimensional structure, while the BIS and ITAQ supported unidimensional models. The BCIS confirmed a two-factor structure (self-reflectiveness and self-certainty), and the SAIQ supported a three-factor model. VAGUS-SR yielded a three-factor structure, while the CR version was unidimensional. Structural properties were not assessed in the EIS, SALI, or Lang et al.’s Index.

#### Construct validity

Construct validity was generally supported through correlations with clinical or functional variables. The MIS, SAI-E, and Schedule for the Assessment of Insight in Psychosis (SIP) showed significant associations with symptom severity and known-group differences. BCIS correlated with SUMD items and cognitive symptoms, and MIC-SR with depression. However, MIC-CR and EIS showed only partial support, with limited or inconsistent correlations.

#### Criterion validity

Formal gold standards were rarely used. ITAQ demonstrated high correlation (*r* = 0.85) with a structured interview. BIS scores aligned with Present State Examination (PSE) insight ratings. SAI-E distinguished involuntary from voluntary admissions. Several scales (e.g. SUMD, SIP, BCIS, and VAGUS) were validated against other established insight measures but not against diagnostic anchors, limiting criterion-level interpretations.

#### Internal consistency

Internal consistency was strong for most scales. Cronbach’s alpha values ranged from 0.71 (MIS positive insight) to 0.92 (SIP). BIS (0.75), AII (0.84), MIC-CR (0.87), and MIC-SR (0.91) exceeded accepted thresholds. Lower consistency was noted in MIS negative insight (0.55) and the BCIS subscales (*α* = 0.60–0.68).

#### Reliability

Reliability was well-established for several tools. SUMD subscales showed Intraclass Correlation Coefficients (ICCs) between 0.79 and 0.90. MIC-CR inter-rater reliability reached *r* = 0.94. SAI-E (ICC = 0.72), BIS (*r* = 0.90 test–retest), MIS-R (ICC = 0.79), and VAGUS-CR/SR (ICC = 0.84–0.99) provided additional evidence. Reliability was not reported for SALI, SAIQ, or the Insight Index scale.

#### Responsiveness

Responsiveness was evaluated in only a few instruments. It refers to a measure’s ability to detect meaningful change over time, particularly in response to clinical improvement or intervention. MIS and BIS demonstrated sensitivity to clinical change, with BIS tracking recovery status and MIS scores improving at discharge (*t* = 3.54, *p* < 0.002). ITAQ scores improved during hospitalization but were not correlated with symptom change. No longitudinal data were reported for VAGUS, SIP, or BCIS, despite their design suggesting potential sensitivity to change.

#### Methodological considerations

Scales varied in their reliance on self-report versus clinician ratings. Discrepancies were noted between formats (e.g. MIC-CR vs. MIC-SR, EIS triangulation), suggesting perspective-dependent ratings. Common limitations included lack of patient input, limited construct coverage, and missing data on test–retest reliability or responsiveness.

#### Cross-study validation synthesis

Due to considerable heterogeneity in study designs, samples, and psychometric approaches, a quantitative meta-analysis was not feasible. Instead, we present a structured narrative synthesis of cross-study validation findings. To complement COSMIN ratings and primary development papers, we integrated evidence from 10 independent validation studies (see Table 2), which offer key data on structural, construct, and criterion validity, as well as reliability, for several widely used insight measures in psychosis.

**Table 2.** Cross-study validation summary

Source	Scale(s) evaluated	Content validity	Structural validity	Construct validity	Criterion validity	Internal consistency	Reliability	Measurement considerations	Methodological considerations
Young et al. (2003)	BIS, SUMD	Assessed self-report versus clinician-report perspective	Not tested	Administration order affected BIS–SUMD correlation (*r* = 0.39 vs. *r* = 0.80)	Differential bias depending on order	Not reported	Modality-specific variability	Order effects suggest interpretive caution	Highlights methodological bias in insight assessment
Pedrelli et al. (2004)	BCIS	Targeted cognitive metacognition in psychosis	Two-factor CFA confirmed: Self-reflectiveness and self-certainty. Fit indices: CFI = 0.96, RMSEA = 0.025	Moderate correlations with BIS and PANSS	No objective criterion standard; clinical convergence	*α* = 0.66 overall; SR = 0.70; SC = 0.55	Acceptable for research use	Validated in the older outpatient population	Cross-sectional; moderate sample size
Greenberger and Serper (2010)	BCIS, SUMD	BCIS captures cognitive insight; SUMD assesses clinical insight dimensions	BCIS: Two-factor model; SUMD structure aligned with prior work	BCIS SR correlated with cognitive symptoms (*r* = −0.42); SUMD with depression (*r* = −0.52)	No gold standard; supported via symptom correlations	BCIS *α* = 0.61–0.84; SUMD *α* = 0.76	Good across psychosis samples	Distinct domains (clinical vs. cognitive insight)	Clarifies theoretical distinction between insight domains
Buchy et al. (2012)	BCIS	Normative study in the general population	Two-factor PCA (self-reflectiveness and self-certainty)	Scores correlated with age and education	No criterion reference; norms only	SR *α* = 0.68; SC *α* = 0.65	Adequate for normative benchmarking	Supports normative use in the general population	Community-based, nonclinical sample
Saperstein et al. (2012)	MIC-CR, MIC-SR	Addresses neurocognitive insight	Not reported	MIC scores linked to symptom severity, not to objective cognitive tests	Low correlation with cognitive testing	*α* = 0.83–0.93	MIC-SR test–retest *r* = 0.92	Measures neurocognitive (versus clinical) insight	Suggests a separate construct for cognitive insight
Michel et al. (2013)	Abbreviated SUMD	The 9-item version retained key insight domains	Three-factor model confirmed. Fit indices: RMSEA = 0.03, CFI = 1.00, GFI = 0.99	Strong correlation with PANSS and G12 (*r* = 0.33–0.67)	PANSS used as external validator	*α* = 0.76–0.83	High across subscales	Suitable for clinical use with linear scoring	Validated in a large multicenter sample (*N* = 531)
Cleary et al. (2014)	BIS	Assesses awareness of illness, relabeling, and treatment need	One-factor model (7 items, after dropping item 1) confirmed in two samples. Fit: CFI > 0.95 (other indices not given)	Moderate convergent validity with symptoms	No criterion standard; clinical correlations used	*α* = 0.80 (7-item version)	Consistent across psychosis samples	Recommend omitting poorly performing item 1	Validated across early and chronic illness stages
Konsztowicz et al. (2018)	SAI-E, BIS, BCIS, SUMD	Combined items from four instruments to analyze multidimensional insight	Five-factor model explained 47.6% variance (e.g. illness awareness, self-certainty)	Confirmed independence of cognitive and clinical domains	No criterion used; factor loadings analyzed	*α* = 0.81 (illness and treatment); others lower (0.44–0.64)	Low for cognitive domains	Supports the use of a multi-method assessment battery	Strong factor analytic design; large sample size
Büchmann et al. (2019)	BIS	BIS was applied in schizophrenia and bipolar I/II groups	Three-factor CFA confirmed: Awareness of illness, relabeling of symptoms, need for treatment. Fit: CFI = 0.953, RMSEA = 0.070	BIS subscales correlated with PANSS, YMRS; lower validity in bipolar II	Not benchmarked to external criteria	*α* = 0.76–0.87	Lower reliability in the bipolar II group	Supports diagnostic comparisons using BIS	Suggests cautious use in bipolar II disorder
Vidal Mariño et al. (2023)	Abbreviated SUMD	Focused on inter-rater reliability using DOMENIC approach	Not evaluated	Assessed item-level agreement by raters	No external validation applied	Not applicable	*κ* = 0.65–0.84; strong for positive, weaker for negative symptoms	An innovative single-patient/multi-rater method was used	Highlights rater drift and the need for rater training

The **SUMD**, especially in its abbreviated form, demonstrated strong psychometric properties. Michel, Baumstarck, Auquier, et al. (2013) confirmed a three-factor structure – awareness of illness and treatment need, awareness of positive symptoms, and awareness of negative symptoms – with excellent model fit (Comparative Fit Index (CFI) = 1.00, Root Mean Square Error of Approximation (RMSEA) = 0.03) and internal consistency (*α* = 0.76–0.83). Criterion validity was supported through significant correlations with Positive and Negative Syndrome Scale (PANSS) General Psychopathology Item 12 (G12) (*r* = 0.67). Inter-rater reliability was further established by Vidal Mariño, Muñoz, Torres, et al. (2023) using the DOuble Measurement with Estimation of Non-Independent Components (DOMENIC) method, revealing high agreement for hallucinations (*κ* = 0.81) and moderate agreement for negative symptoms (*κ* = 0.65).

The **BIS** was supported by both unidimensional and multidimensional models. Cleary, Shapiro, Jackson, and Heinssen (2014) validated a seven-item, one-factor structure across first-episode and chronic illness samples. Büchmann, Wirtz, Köhler, and Wiedemann (2019) confirmed a three-factor structure – awareness of illness, relabeling of symptoms, and need for treatment – with acceptable model fit (CFI = 0.92, RMSEA = 0.070) in schizophrenia and bipolar I samples. Convergent validity was supported by moderate to strong correlations with observer-rated insight (*r* = −0.49 to −0.55), although performance was weaker in bipolar II.

Modality-specific biases were reported in studies comparing clinician-rated and self-report instruments. Young, Finnerty, and Gerson (2003) found that BIS and SUMD correlations varied significantly depending on administration order (*r* = 0.39 vs. 0.80), suggesting method variance. Konsztowicz, Lachance, Joober, and Malla (2018) also compared items from SAI-E, BIS, BCIS, and SUMD in a joint factor model, identifying five insight dimensions that spanned clinical and cognitive domains. Reliability was high for ‘Illness and Treatment’ (*α* = 0.81), but lower for cognitive subdomains (*α* = 0.44–0.64).

The **BCIS** consistently demonstrated a two-factor structure (self-reflectiveness and self-certainty) across populations. Self-reflectiveness was inversely correlated with cognitive symptoms (*r* = −0.42), and self-certainty was positively associated with impaired insight (Greenberger & Serper, 2010; Pedrelli, McQuaid, Granholm, & Jeste, 2004). Internal consistency ranged from *α* = 0.60 to 0.84. Buchy, Brodeur, and Lepage (2012) validated the scale in a Canadian general population sample, confirming the factor structure and identifying demographic influences.

The **MIC**, assessed by Saperstein et al. (2012), showed excellent internal consistency (*α* = 0.83–0.93) and test–retest reliability (MIC-SR *r* = 0.92). While MIC scores were not correlated with neuropsychological test performance, they were significantly associated with symptom severity, underscoring their clinical relevance in evaluating neurocognitive insight. Collectively, these10 studies affirm the multidimensional nature of insight and the importance of combining clinician- and self-report instruments. They also highlight both the strengths and limitations of available tools across settings and populations.

## Discussion

Our aim in this study was to evaluate the quality of the psychometric properties of insight scales developed to measure lack of insight in psychotic disorders. We conducted a systematic and comprehensive search spanning back to the early twentieth century, when the concept of insight in psychosis first became formally articulated (Lewis, 1934). The initial screening yielded over 2,000 publications; following full-text review, we identified 15 distinct insight scales and 10 studies that specifically evaluated the psychometric performance of one or more of these instruments.

Reviewing a century of research revealed that, for most of the twentieth century, insight into psychosis was assessed in a largely impressionistic manner (Supplementary Appendix 1). Although attempts to quantify the lack of insight can be found as early as the 1950s and 1960s, it was not until the late 1980s that the first structured insight scales – those with reportable psychometric properties – were developed.

### Summary of psychometric findings

Across the 15 identified insight scales, content validity was generally adequate to very good, with several instruments (e.g. SUMD, BIS, MIS, MIS-R, BCIS, MIC, and VAGUS) explicitly grounded in established theoretical models and demonstrating comprehensive coverage of key insight domains. Structural validity varied: some scales (e.g. BIS, BCIS, MIC, and MIS) were supported by clear factor analytic evidence, whereas others (e.g. SALI, Lang Index, and EIS) lacked empirical evaluation of dimensionality.

Construct validity, most often assessed through hypothesis testing and convergent correlations with related measures (e.g. symptom severity, illness awareness, and treatment adherence), was supported for the majority of scales. Known-groups validity was also demonstrated in several cases, such as lower insight scores in involuntary versus voluntary admissions (SAI-E) and higher scores in recovered versus non-recovered patients (BIS). Criterion validity, in the strict sense of comparison to a gold standard, was largely absent given the lack of a universally accepted benchmark for insight, although many scales showed strong convergence with widely used comparator measures, such as the SAI, BIS, and SUMD.

Internal consistency ranged from acceptable to excellent in most instruments, with Cronbach’s alpha often exceeding the COSMIN threshold of 0.70. Reliability evidence was more variable: while measures such as the MIS-R, BIS, AII, MIC, and VAGUS demonstrated good inter-rater or test–retest reliability, others lacked formal reproducibility data. Responsiveness to change – a critical property for clinical and longitudinal research – was seldom assessed, with notable exceptions being the BIS and MIS, which showed sensitivity to clinical improvement over time.

Taken together, these findings suggest that although insight measurement in psychosis has advanced considerably since the late 1980s, gaps remain in the evaluation of structural validity, reproducibility, and responsiveness for several widely used instruments.

### Methodological considerations: Modality, bias, and utility

Marked methodological differences emerged between clinician-rated and self-report insight scales. Across multiple studies, self-report measures typically yielded lower insight scores than clinician-administered tools (Konsztowicz et al., 2018; Young et al., 2003), a discrepancy likely reflecting differences in perspective, the influence of social desirability, and genuine metacognitive deficits (Tranulis, Corin, & Kirmayer, 2008a; Tranulis, Lepage, & Malla, 2008c). Such modality-based variation underscores the importance of multi-informant approaches and cautions against over-reliance on any single method. Order effects in test administration may further bias results (Young et al., 2003).

Clinician-rated insight scales can incorporate observational data and clinical history but may be susceptible to the rater’s bias and may underrepresent the patient’s subjective experience (Amador & David, 2004). Self-report scales are efficient and straightforward to administer, yet their validity can be undermined by response biases such as social desirability or cognitive limitations (Beck et al., 2004; Saperstein et al., 2012). They often produce lower scores than clinician ratings, cover fewer insight dimensions, and have weaker evidence for reproducibility and responsiveness (Konsztowicz et al., 2018; Young et al., 2003), limiting their utility as stand-alone tools.

Hybrid formats, such as the VAGUS or Insight Index, show promise in integrating perspectives and addressing the limitations of single-modality assessment. Practical factors, such as administration time, scoring complexity, and training, need further influence selection: while the SUMD provides a detailed multidimensional assessment, it is resource-intensive and less feasible for routine care, whereas brief tools, such as the BIS or BCIS, offer efficiency at the expense of contextual depth.

### Cross-cultural applicability and generalizability

Despite widespread use, most scales were developed and validated in North American or European contexts. Only a few, such as the SIP (Yen, Yeh, Chong, Chung, & Chen, 2001) and the Insight Index (Lang et al., 2003), emerged from non-Western settings. Moreover, none of the instruments in this review were subjected to formal cross-cultural adaptation using methods such as measurement invariance testing.

This lack of cultural validation limits the interpretability of insight scores across diverse populations and potentially overlooks culturally embedded understandings of illness and treatment. For instance, explanatory models of psychosis may differ significantly across regions, influencing how insight is expressed and perceived (Chakraborty & Basu, 2010). Future research must address this gap by developing or adapting instruments for use in underrepresented settings.

### Directions for future research and clinical use

Future work should address persistent gaps in psychometric evaluation, particularly structural validity, reproducibility, and responsiveness. Importantly, the relevance of responsiveness depends on the conceptual model underpinning each scale: some measures aim to capture relatively stable metacognitive traits, while others are designed to track changes in clinical status. Applying responsiveness criteria indiscriminately risks conflating different and distinctly valuable goals.

Improved cross-cultural validation, including translation, cultural adaptation, and measurement invariance testing – remains a priority. The use of multimodal assessment, combining clinician ratings, self-report, and collateral perspectives, is likely to yield the most comprehensive evaluation of insight. Instruments, such as the VAGUS, BIS, and BCIS, illustrate efforts to balance theoretical depth, psychometric strength, and clinical feasibility, yet the construct of insight remains multifaceted and context dependent. Measurement may also need to embrace conceptual pluralism about insight, which in each distinct approach is manifested in methodological rigor, theoretical coherence, and adaptability to diverse clinical and cultural contexts.

Finally, incorporating metacognitive constructs into the definition of insight introduces additional conceptual complexity. While such expansion can enrich theoretical models and broaden research engagement, it is important to recognize that cognitive insight and traditional clinical insight are only modestly correlated (*r* = 0.16; Nair et al., 2014), and patients with good cognitive insight do not necessarily demonstrate good clinical insight (Donohoe et al., 2009). Thus, selecting an insight scale requires conceptual clarity and alignment with the evaluator’s aims, the dimension of insight assessed, and the clinical or research context to ensure valid interpretation and maximize its utility.

## Supporting information

Hazan et al. supplementary material Hazan et al. supplementary material

## Supplementary material

The supplementary material for this article can be found at http://doi.org/10.1017/S0033291725101918.
