# Supplementary material for: Instruments for assessing insight in psychosis: A systematic review of psychometric properties
Source: Psychol Med. 2025 Nov 26;55:e362. doi: 10.1017/S0033291725101918 (PMC12671917; doi:10.1017/S0033291725101918)
Supplement: Hazan et al. supplementary material [file S0033291725101918sup001.zip › S0033291725101918sup001.docx]

Appendix 5. Detailed Psychometric Properties of Insight Scales (n = 15)

| Scale | Content Validity | Structural Validity | Construct Validity | Criterion Validity | Internal Consistency | Reliability | Responsiveness |
| --- | --- | --- | --- | --- | --- | --- | --- |
| Davidhizar (1986) | Developed from prior literature; no patient input. | No factor analysis reported. | Correlated with attitude scales and inversely with hallucinations. | No gold standard comparison. | Cronbach’s α = 0.903. | No test–retest or inter-rater data reported. | Not reported. |
| ITAQ (1989) | Expert-rated; no patient involvement. | PCA supported unidimensional structure. | Moderate correlation with BPRS, CGI, and medication adherence. | High correlation (r = 0.85) with open interview (not a validated standard). | Not reported. | Inter-rater reliability of open interview (r = 0.82); no ITAQ-specific data. | Change observed from admission to discharge, but not anchored to external criteria. |
| SAI/SAI-E (1990/92) | Theory-based domains (awareness, relabeling, compliance). | PCA showed a single-factor solution explaining 60.2% of variance. | Correlated with IQ, psychopathology, PSE; lower in involuntarily admitted patients. | No gold standard used. | High inter-item correlations (r = 0.50–0.75); alpha not reported. | Inter-rater ICC = 0.72. | Not tested longitudinally. |
| MIS/MIS-R (1992/03) | Theory-informed items; revised for clarity. | EFA supported multidimensional structure: 5 (positive), 3 (negative); MIS-R yielded 4 factors (48.3% variance). | Correlated with symptom severity and insight measures; MIS-R correlated with PSE insight rating. | No formal gold standard used. | MIS: α = 0.71 (positive), α = 0.55 (negative); MIS-R α = 0.875. | MIS-R test–retest r = 0.65; ICC = 0.79. | MIS showed improvements from admission to discharge. |
| SUMD (1993) | Multidimensional model; structured for awareness and attribution (current/past). | No factor analysis; subscales treated as independent. | Correlated with insight measures; poorer insight linked to poorer adherence and illness course. | No formal gold standard. | Not calculable due to hierarchical structure. | Inter-rater ICCs = 0.79–0.90. | Not assessed. |
| BIS (1994) | Based on three-component model; piloted for clarity. | One-factor structure accounting for 60.4% of variance. | Differentiated groups via PSE; correlated with insight dimensions (r = 0.33–0.56). | PSE used as external reference; not a validated standard. | α = 0.75; moderate inter-item correlation. | Test–retest: total r = 0.90; subscales r = 0.65–0.96. | Sensitive to change; tracked recovery status. |
| AII (1996) | Face-valid items addressing illness recognition and treatment need. | 2-factor structure explaining 69% of variance. | Correlated with baseline adherence; not predictive longitudinally. | No formal gold standard. | α = 0.84 (total); α = 0.75 (subscales). | Inter-rater ICC > 0.75. | Not evaluated for responsiveness. |
| SALI (1998) | Clinically informed items; no formal pilot testing. | No dimensionality analysis; structural validity unclear. | Correlated with PANSS items; hypotheses not pre-specified. | Compared to PANSS G12; not a gold standard. | α = 0.85; dimensionality unclear. | Single-rater only; no reliability testing. | Not assessed. |
| SAIQ (2000) | Adapted from PEH; meaningful domains included. | PCA identified 3-factor structure explaining 56.3% of variance. | Strong correlations with SUMD and PANSS G12. | No gold standard, but convergent evidence strong. | α = 0.83 (total); subscales α = 0.86, 0.77, 0.72. | No reliability data reported. | Not evaluated longitudinally. |
| SIP (2001) | Multidimensional model including symptoms, relapse, and life changes. | No factor analysis reported; domains conceptually distinct. | High correlations with SUMD and SAI (r = 0.75–0.90). | Validated against SUMD and SAI. | α = 0.92 (total); subscales = 0.71–0.83. | No inter-rater data reported. | Not assessed. |
| Lang et al. (2003) | Conceptual model; no formal review or structure testing. | No factor analysis; structure assumed. | Insight scores associated with treatment value, hospitalization history. | No gold standard or criterion validation. | No reliability or alpha data reported. | Inter-rater reliability not statistically reported. | Not assessed. |
| BCIS (2004) | Items derived from clinical theory and metacognition literature. | PCA revealed two factors: Self-Reflectiveness and Self-Certainty. | Correlated with SUMD items; differentiated psychosis diagnosis. | Validated against SUMD. | α = 0.68 (Self-Reflectiveness), α = 0.60 (Self-Certainty). | Self-report format; inter-rater reliability not applicable. | Responsiveness not evaluated. |
| MIC-CR (2008)  MIC-SR (2008) | Theory-informed; clinician consensus and patient pilot tested. | Three-factor model; first factor explained 41% variance. | Modest correlation with affective symptoms; not correlated with PANSS G12. | Criterion validity not assessed. | α = 0.87. | Inter-rater r = 0.94. | Not evaluated. |
|  | Derived from MIC-CR with patient-friendly language. | Two-factor model; primary factor = 51.6% variance. | Correlated with PANSS depression; not with PANSS G12. | Criterion validity not assessed. | α = 0.91; item removal had minimal impact. | Inter-rater reliability not applicable. | Not evaluated. |
| EIS (2008) | Based on prior instruments; scored via qualitative narrative coding. | No dimensionality analysis; domains treated as theoretically derived. | Correlated with family member scores (r = 0.51); no clinician agreement. | No gold standard; qualitative validity focus. | Internal consistency not calculated (dichotomous coding). | Inter-rater κ = 0.63. | Not assessed. |
| VAGUS (2014) | Derived from validated tools (SAI, BIS, SUMD); piloted for self- and clinician formats. | CR: 1-factor (53.8% variance); SR: 3-factors (63.1%). | Strong correlations with SAI and BIS; low BPRS correlations. | No gold standard, but strong convergence supports criterion-related inference. | α = 0.745 (CR); α = 0.773 (SR). | ICC = 0.99 (CR); test–retest = 0.84 (CR), 0.91 (SR). | Not assessed; designed for change sensitivity but untested. |
