# Supplementary material for: Instruments for assessing insight in psychosis: A systematic review of psychometric properties
Source: Psychol Med. 2025 Nov 26;55:e362. doi: 10.1017/S0033291725101918 (PMC12671917; doi:10.1017/S0033291725101918)
Supplement: Hazan et al. supplementary material [file S0033291725101918sup001.zip › S0033291725101918sup002.docx]

**Appendix 1**. Review of impressionistic insight assessment

| Authors | Year | Study Aim | Sample Size | Study Population | Theoretical Definition of Insight | Operational Definition of Insight | Item Number | Measured Factors | Scoring Method | Study Outcome |
| --- | --- | --- | --- | --- | --- | --- | --- | --- | --- | --- |
| Eskey | 1958 | To explore whether psychotic patients with insight into their illness have a better prognosis, defined as shorter hospitalization, compared to those with partial or no insight. | 300 psychotic patients divided into three groups: insight (N=100), partial insight (N=100), no insight (N=100) | Psychotic patients (age 15-70) admitted to a psychiatric hospital in last 2 weeks | verbal recognition by the patient of existing psychological difficulties | Verbalized statements that reflect an awareness of malfunctioning on their part (e.g., I seem confused, something is wrong with my mind) | N/A | Length of hospitalization, level of insight (full, partial, none) | The presence or absence of insight was determined through an evaluation of each patient’s mental status examination | Patients with insight did not improve more rapidly (as evaluated by the number of days of hospitalization) than patients without insight |
| Whitman & Duffey | 1961 | To examine how different treatment modalities (chemotherapy alone vs. chemotherapy combined with psychotherapy) affect schizophrenic patients' perception of their illness and hospitalization. | 24 for chemotherapy group; 19 for chemotherapy + psychotherapy group | Newly admitted schizophrenic patients; chronic schizophrenic patients | Insight is the patient's understanding and acceptance of their hospitalization being due to a psychological or functional disorder. | Insight is operationalized as the patient's ability to provide "functional" reasons for their own hospitalization, implying they recognize their psychiatric condition. | 8 critical questions in the questionnaire | Self-perception of hospitalization reason (functional vs. non-functional) | Verbatim responses scored by two clinical psychologists based on whether the reasons given were psychological ("functional") or non-functional (e.g., organic illness, no reason) | Chemotherapy group showed decreased insight after 4 weeks, with fewer patients providing functional reasons for their hospitalization. The psychotherapy group maintained insight, indicating the importance of psychotherapy in fostering understanding of mental illness. |
| Soskis & Bowers | 1969 | To assess attitudes and (3 to 7 years) post-hospital adjustment in former schizophrenic patients | 32 | Former schizophrenic patients from Yale-New Haven Hospital | The degree to which patients recognized their illness and were able to reflect on it. | Attitude questionnaire utilizing positive/negative and insight-related attitudes | 10 |  | The scoring method was based on the degree to which patients endorsed various attitudes about their illness and recovery. Scores were calculated for each attitude, and higher scores on positive attitudes (e.g., positive insight) were associated with better post-hospital adjustment, while higher scores on negative attitudes (e.g., negative insight) correlated with poorer outcomes. | Positive attitudes were linked to better adjustment post-hospitalization; negative insight attitudes correlated with symptom discomfort and poorer outcomes |
| Kennard | 1974 | To explore discrepancies between patients’ self-perception on admission and perceptions by closest others (COPs). | 20 patients and 20 COPs | Newly admitted psychiatric patients and the individuals most involved in their admission (family members or close contacts). | Ability to perceive one's psychological state compared to how they believe they should be and how they perceive mental illness. | Self-assessment by patients compared with their ideal state and their view of a mentally ill person. | 10 | Patients' and COPs' views on disturbed behavior, communication, illness, and social factors. | Semantic differential method using Osgood’s factors (Evaluative, Activity, Potency), patient-COP discrepancies analyzed through correlation and factor analysis. | Significant discrepancies between patients' and COPs' perceptions of disturbed behavior, communication, and illness. Patients saw themselves closer to ideal, while COPs did not align clearly. |
| Van Putten, Crumpton & Yale | 1976 | To explore why some chronic schizophrenic patients refuse medication and prefer psychosis. | 59 | Chronic schizophrenic patients, divided into drug-refusers and drug-compliers. | Patients' ability to recognize their illness and need for medication. | Insight was rated as "yes" if the patient acknowledged some awareness of emotional illness; "no," if patient vigorously denied the fact that he was disturbed. | N/A | Grandiosity, depression, anxiety, drug refusal, insight into illness, social isolation, global assessment at discharge. | Patients classified as drug-refusers or drug-compliers, with ratings for grandiosity, anxiety, depression, and insight. Discriminant function analysis used to identify predictors. | Drug-refusers had less insight, more grandiosity, and more involuntary hospitalizations. Grandiosity was the strongest predictor of drug refusal. Drug-compliers had more anxiety and depression. |
| Roback & Abramowitz | 1979 | To examine the relationship between insight and adjustment in schizophrenics | 24 | male patients from both open and closed wards  hospital. All but four were diagnosed as schizophrenic. | Not explicitly defined, but implied to be related to understanding of one's own and others' motivations and behaviors | Scores on the Tolor-Reznikoff Test | 27 | Social insight (as a proxy for self-insight) | Multiple-choice test with four alternative interpretations for each social situation. Scores were ranked and trichotomized to form high and low insight groups. | Patients with higher insight scores were rated as better adjusted behaviorally by hospital staff but reported more psychological distress compared to those with lower insight scores. The authors suggest that insight in schizophrenics may intensify subjective distress while being conducive to behavioral adaptation. |
| Appelbaum, Mirkin & Bateman | 1981 | To assess competency to consent to psychiatric hospitalization in newly admitted patients. | 50 | Newly admitted psychiatric patients | Awareness of psychiatric condition and need for treatment. | Responses to questions about psychiatric awareness and need for hospitalization. | 15 | Competency to consent to hospitalization, awareness of psychiatric condition, treatment needs, legal rights. | Responses scored on a 3-point scale (0 = unacceptable, 1 = partially acceptable, 2 = fully acceptable). | Less than half of patients showed clear awareness of psychiatric problems or need for hospitalization. Significant gaps in understanding legal rights, highlighting impairments in competency. |
| McEvoy, Wilson, & Hawkins | 1981 | To assess chronic schizophrenic inpatients' understanding of their illness, need for admission, and medication | 45 | Chronic schizophrenic inpatients at an extended treatment program in Nashville | Awareness of having an emotional or mental illness and recognizing the need for medical intervention. | Insight was scored based on patient responses to questions about their illness, hospitalization, current state, and medication needs. Scores reflected "good," "fair," or "poor" insight depending on the level of awareness expressed. | 11 | Understanding of illness, need for admission, current state, medication awareness, misconceptions about medication | Responses scored by a clinical psychologist on a scale: good (1), fair (0.5), or poor (0). The total score ranged from 0 to 11. | Most patients showed low insight levels: only 13% understood they were mentally ill, and 27% recognized a need for medication. Scores on insight were not correlated with improvement in medication response. Many lacked sufficient insight to make sound decisions about medication compliance. |
| Greenfeld, Strauss, Bowers, Mandelkern | 1989 | To explore the dimensions of insight in patients recovering from psychosis | 21 | Patients recovering from acute psychosis at Yale-New Haven Hospital | Insight is viewed as multidimensional, involving: (1) views on symptoms, (2) illness acknowledgment, (3) beliefs about causes (etiology), (4) vulnerability to recurrence, and (5) opinions about treatment | Insight assessed via semi-structured interviews, focusing on five dimensions: symptom views, acknowledgment of illness, causal beliefs, vulnerability to relapse, and views on treatment effectiveness | 5 | Symptom views, illness existence, causal beliefs, relapse vulnerability, treatment value | Semi-structured interview responses were categorized across five main dimensions of insight | Patients displayed a range of insights from denial to complex, detailed understanding; insight varied in coherence, and collaborative treatment was linked to patients' views on relapse vulnerability and causes of their psychosis. |
