# Supplementary material for: Instruments for assessing insight in psychosis: A systematic review of psychometric properties
Source: Psychol Med. 2025 Nov 26;55:e362. doi: 10.1017/S0033291725101918 (PMC12671917; doi:10.1017/S0033291725101918)
Supplement: Hazan et al. supplementary material [file S0033291725101918sup001.zip › S0033291725101918sup004.docx]

**Appendix 4.** List of papers extracted for review (n = 28)

|  | **Year** | **First Author** | **Title** | Present a new instrument | Comment |
| --- | --- | --- | --- | --- | --- |
|  | 1986 | Davidhizar | Insight Instrument | ✔️ |  |
|  | 1989 | McEvoy | Insight In Schizophrenia. Its Relationship To Acute Psychopathology | ✔️ |  |
|  | 1990 | David | Schedule For Assessing The Three Components Of Insight | ✔️ |  |
|  | 1992 | David | The Assessment Of Insight In Psychosis | ✔️ |  |
|  | 1992 | Markova | The assessment of insight in clinical  psychiatry: a new scale | ✔️ |  |
|  | 1993 | Amador | Assessment Of Insight In Psychosis | ✔️ |  |
|  | 1994 | Birchwood | A Self-Report Insight Scale For Psychosis - Reliability, Validity And Sensitivity To Change | ✔️ | A self-report Insight Scale  is presented |
|  | 1996 | Cuffel | Awareness Of Illness In Schizophrenia And Outpatient Treatment Adherence | ✔️ | Awareness of Illness Interview |
|  | 1998 | Debowska | Insight in Paranoid Schizophreniamlts Relationship  to Psychopathology and Premorbid Adjustment | ✔️ | Paranoid schizophrenia patients in Poland |
|  | 2000 | Marks | Self-Appraisal of Illness Questionnaire (SAIQ):  relationship to researcher-rated insight and neuropsychological  function in schizophrenia | ✔️ | The Self-Appraisal of Illness Questionnaire (SAIQ) is a self-report instrument designed to assess attitudes toward  mental illness among persons receiving psychiatric treatment |
|  | 2001 | Yen | A Multidimensional Assessment Of Insights In Schizophrenic Patients | ✔️ | Scale to assess multidimensional insight in schizophrenic patients |
|  | 2002 | Lang | Predictors Of Insight Into Mental Illness Among Psychotic And Non-Psychotic Patients | ✔️ | index of insight into mental illness |
|  | 2003 | Markova | Assessment Of Insight In Psychosis: A Re-Standardization Of A New Scale | ✔️ |  |
|  | 2004 | Beck | A New Instrument For Measuring Insight: The Beck Cognitive Insight Scale | ✔️ |  |
|  | 2008 | Medalia | Insight Into Neurocognitive Dysfunction In Schizophrenia | ✔️ | Uses the Measure of Insight into Cognition—Self Report (MIC-SR), which is a self-report measure assessing awareness of cognitive difficulties. |
|  | 2008 | Medalia | Do People With Schizophrenia Who Have Objective Cognitive Impairment Identify Cognitive Deficits On A Self Report Measure? | ✔️ | Measure of Insight into Cognition—Clinician Rated (MIC-CR), which is a clinician-rated scale that evaluates both awareness and attribution of cognitive deficits. |
|  | 2008 | Tranulis | Insight And Psychosis: Comparing The Perspectives Of Patient, Entourage And Clinician | ✔️ | Extracted Insight Scale was designed to measure the general level of insight based on the dimensions of insight most commonly found in the literature. |
|  | 2014 | Gerretsen | The Vagus Insight Into Psychosis Scale - Self-Report And Clinician-Rated Versions | ✔️ |  |
|  | 2004 | Pedrelli | Measuring Cognitive Insight In Middle-Aged And Older Patients With Psychotic Disorders | ❌ | test psychometric properties, factor structure and validity of BCIS |
|  | 2003 | Young | A Comparison Between An Interview And A Self-Report Method Of Insight Assessment In Chronic Schizophrenia | ❌ | To examine the relationship between a researcher-rated and a self-report method SUMD and IS) of insight assessment in patients with schizophrenia |
|  | 2010 | Greenberger | Examination Of Clinical And Cognitive Insight In Acute Schizophrenia Patients | ❌ | \| To investigate the clinical utility and coherence of the BCIS in acute schizophrenia patients. \| \| --- \|  \|  \| \| --- \| |
|  | 2012 | Saperstein | The Measure Of Insight Into Cognition: Reliability And Validity Of Clinician-Rated And Self-Report Scales Of Neurocognitive Insight For Schizophrenia | ❌ | To evaluate the reliability and validity of clinician-rated and self-report measures of **neurocognitive insight**. |
|  | 2012 | Buchy | The Beck Cognitive Insight Scale: Psychometric Properties In A Canadian Community Sample | ❌ | To assess the psychometric properties of the BCIS in a Canadian community sample. |
|  | 2013 | Michel | Psychometric Properties Of The Abbreviated Version Of The Scale To Assess Unawareness In Mental Disorder In Schizophrenia | ❌ | To evaluate the validity and reliability of the abbreviated version of the SUMD in patients with schizophrenia. |
|  | 2014 | Cleary | Measuring Insight Through Patient Self-Report: An In-Depth Analysis Of The Factor Structure Of The Birchwood Insight Scale | ❌ | validating the Birchwood Insight Scale (BIS) |
|  | 2015 | Konsztowicz | Dimensions Of Insight In Schizophrenia: Exploratory Factor Analysis Of Multiple Self- And Interviewer-Rated Measures Of Insight | ❌ | To identify the dimensions of insight in schizophrenia through factor analysis of existing measures. |
|  | 2019 | Buchmann | Validity Of The Birchwood Insight Scale In Patients With Schizophrenia Spectrum- And Bipolar Disorders | ❌ | To evaluate the validity of the Birchwood Insight Scale in assessing insight among patients with schizophrenia spectrum disorders. |
|  | 2023 | Marino | Evaluating The Inter-Rater Reliability Of The Scale To Assess Unawareness Of Mental Disorder Using The Domenic Method | ❌ | \| To evaluate the inter-rater reliability of the Scale to Assess Unawareness of Mental Disorder using the DOMENIC method. \| \| --- \|  \|  \| \| --- \| |
