# Supplementary material for: Instruments for assessing insight in psychosis: A systematic review of psychometric properties
Source: Psychol Med. 2025 Nov 26;55:e362. doi: 10.1017/S0033291725101918 (PMC12671917; doi:10.1017/S0033291725101918)
Supplement: Hazan et al. supplementary material [file S0033291725101918sup001.zip › S0033291725101918sup005.pdf]

Search Strategy for Systematic Review on Insight in Psychosis

This document outlines the detailed search strategy for the systematic review on the measurement properties of instruments used to assess insight in psychosis. The search strategies are tailored to each database to ensure comprehensive and systematic identification of relevant literature.

Ovid MEDLINE ALL Search Strategy

1. exp psychotic disorders/ or exp Schizophrenia/
2. *Awareness/ or *Attitude/ or *"Attitude to Health"/
3. exp "Surveys and Questionnaires"/ or exp Interview Psychological/ or exp Psychiatric Status Rating Scales/
4. ((psychosis or psychoses or psychotic or schiz*) adj7 (insight* or awareness or attitude* or belief* or perception or self-awareness or self-understanding or cognizan* or realization or intuition or discernment or grasp or perceptive or perceptiveness or savvy or unawareness or comprehension)).twkw.
5. exp "Surveys and Questionnaires"/ or exp Interview Psychological/ or exp Psychiatric Status Rating Scales/ or (assessment* or checklist* or check list* or instrument* or inventor* or measure* or questionnaire* or scale or scales or score or scores or survey or surveys or test or tests or tool or tools or interview*).twkw.
6. "Reproducibility of Results"/ or Psychometrics/ or (reliability or reliabl* or validate$ or validation or validity or psychometric* or responsiv* or interpretab* or "measurement error*" or "error of measurement*" or "errors of measurement*" or consistency or "factor analys*" or "factor structure" or "item analys*" or "item response" or "item functioning" or "component analys*" or "measurement invariance" or "test construction" or sensitivity or specificity or "statistical rotation" or structured or semistructured or "oblique rotation" or "orthogonal rotation" or "varimax rotation" or "split half" or "test retest" or interrater or "inter rater" or intrarater or "intra rater" or stability or equivalence or "intraclass correlation coefficient*" or "intra-class correlation coefficient*" or kappa or "coefficient of correlation" or "coefficients of correlation" or "correlation coefficient*" or "pearson product moment" or "spearman rho" or "minimal detectable difference" or "minimal detectable change").twkw.
7. 2 and 3 and 4
8. 1 or 5
9. limit 6 to english language
